# ESTSS at 20 years: “a phoenix gently rising from a lava flow of European trauma”

**DOI:** 10.3402/ejpt.v4i0.21306

**Published:** 2013-06-06

**Authors:** Roderick J. Ørner

**Affiliations:** Visiting Professor, Faculty of Health, Life and Social Sciences, University of Lincoln, Lincoln, UK; Clinical Psychologist in Private Practice Specialising in Maritime Trauma (ForceMajeureMaritime.com)

**Keywords:** ESTSS, psychotraumatology, foundation, memory, ethics, human rights

## Abstract

Roderick J. Ørner, who was President between 1997 and 1999, traces the phoenix-like origins of the European Society for Traumatic Stress Studies (ESTSS) from an informal business meeting called during the 1st European Conference on Traumatic Stress (ECOTS) in 1987 to its emergence into a formally constituted society. He dwells on the challenges of tendering a trauma society within a continent where trauma has been and remains endemic. ESTSS successes are noted along with a number of personal reflections on activities that give rise to concern for the present as well as its future prospects. Denial of survivors’ experiences and turning away from survivors’ narratives by reframing their experiences to accommodate helpers’ theory-driven imperatives are viewed with alarm. Arguments are presented for making human rights, memory, and ethics core elements of a distinctive European psycho traumatology, which will secure current ESTSS viability and future integrity.

While the foundation of International Society for Traumatic Stress Studies (ISTSS), formerly the Society for Traumatic Stress Studies (STSS) when its international remit had not fully been taken into account, has its roots in wars fought in S.E. Asia, the European Society for Traumatic Stress Studies (ESTSS) arises from an altogether different legacy. Its heritage is so different that the existence of our society is a remarkable achievement against all odds of time. Its origins and survival bear witness to an organisational resilience nurtured from a resolve to minimise exclusivity, avoid doctrinaire orthodoxies and always look ahead with a keen eye to successors who will carry the society forward. The ESTSS has earned a special reputation of tolerance and optimism within its embrace of human relationships. I hope this will be its future too.

The ESTSS grew out of a unique time-specific context in modern European history. During the 1980s, the continent was characterised by an unprecedented matrix of social, military, and political influences, the likes of which had never existed before and could be found nowhere else. Neither specific wars nor critical events gave rise to calls for a European society dedicated to traumatic stress studies. Yet, in its founding stages the embryonic organisation was nurtured by a resonant zeitgeist (recognition of child abuse and domestic violence) which over 20 years has been further strengthened by the ESTSS. The legacy of our society is therefore a timeline that stretches back into a distant past. Virtually all annals of European history carry recurrent themes of trauma, trauma resilience, and survival.

## Roots of the ESTSS

Estimated figures for total numbers of people killed during World War II are incomprehensible in scale. Consider too that among the survivors of this killing spree, 15 million were refugees in the year after its ending. Yet Europe has regenerated itself with new social and political purpose fostered by an unprecedented sense of international tolerance, understanding and appreciation of how we are all dependent upon one another. However, before this positive focus had taken root, Europeans lived with trauma and threat of nuclear annihilation as endemic features of their lives. Historically speaking, trauma was not and had not been viewed as extraordinary.

So, when some of us sought to draw attention to compellingly interesting aspects of trauma and traumatic stress reactions, others wondered what the point would be. We were chastened on being reminded that trauma in European culture and history is not as exceptional as often assumed. To turn our expertise and instruments of systematic investigation on to trauma was a bit like suggesting fish should have a society for studying water or birds an organisation for researching air.

But the most commonplace is sometimes the most interesting and our interest won the day, thanks to all those who became its founding members and attended our first, second, and third European conferences. Together we have led the way and succeeded in waking Europe up to the distinctiveness of trauma, its rightful place as a subject of methodical study and the ways its sequelae are amenable to helpful interventions. Resignation in the face of trauma is no longer the modus operandi in Europe. The phoenix did rise and is still on the wing, if not exactly soaring!

## Resisting the allure of a return to ashes

For all its commendable achievements, the ESTSS with its phoenix eye perspective should be watchful of its onward flight. In Greek mythology, phoenixes arise from the ashes of their predecessors and do so in an endlessly repetitive cycle of destruction and re-generation. Professor Weisæth and Sund (1982) illustrated this dynamic in early reviews of military psycho-traumatology. Lessons learned about informed survivor care were lost to memory in the recurrent cycles of crises (World War I) being followed by temporary resolutions (The Versailles Treaty of 1918) until trauma erupted once more (World War II). Also, battle trauma was construed as a specialist interest with scant clinical or theoretical relevance.

The notion that insights about trauma can be applied in general clinical practice did not take hold until recent decades. To his credit, Freud tried to do so by pointing to evidence of child sexual abuse. But under pressure from those who did not wish to know the truth, he changed focus. Reality became fantasy, except for the victims (Masson, 1984).

Thankfully for the emergent ESTSS, professional, social, and political changes in Europe after 1945 brought greater openness about that which had previously been hidden. Inevitably, the spectres of racial, domestic, sexual, and interpersonal violence would be recognised as matters of public concern.

## From modest beginnings to a self-consciously European ambition

My recollection upon organising the First European Conference on Traumatic Stress in Lincoln, UK, in August/September 1987, with support from my local National Health Service (NHS) employer and the British Psychological Society, is that I had no thoughts of establishing an ESTSS. Thankfully, some conference delegates arrived with other ideas. History shall record that Dr. Stuart Turner was constantly on my back during the first days of the gathering insisting that we should call a business meeting. And so it came to be. We had no formal agenda for our meeting yet the question on everyone's mind was whether to establish a European network or join the STSS.

The matter was settled without a formal motion or vote. The decisive moment occurred when an American colleague made a long, impassioned plea for joining the STSS. Inducements included having access to US expertise and being granted opportunities to build further upon pioneering endeavours across the Atlantic. With such advocacy in full, a consensus emerged that we would start work on our ESTSS. To this end, the business meeting sanctioned the formation of a working group of four volunteer members.

## Taking off on snipped wings

Steered along by Dr. S. Turner, a group comprising of among others, Jos Weerts, Atle Dyregrov, Wolter de Loos and myself moved plans forward for a nascent ESTSS. Members of the working group had to run the gauntlet of the STSS board when the groups met during the World Congress of Psycho-traumatology in Amsterdam during the spring of 1992. Our progress toward founding a European society was perceived by some STSS board members as an attack on the US collegiate. Our local initiative was construed as competitive, based on weak foundations and eventually we would come to realise the error of our ways.

In Amsterdam, the ethos of trauma engendered largely on other continents became a manifest in our first cautious intercontinental negotiations. I recall disbelief during the meeting and emerged “shell shocked”. Rather naively perhaps, thoughts that we may provoke the ISTSS had not occurred to us, but we were in no way deterred from persevering with our European mandate.

Our confidence in having embarked on a sensible course of development with a European focus was reinforced with support given to the 2nd and 3rd European conferences. It was during the latter that the ESTSS was formally constituted and Dr. W.S. de Loos was elected its first President backed by a secretariat in the Netherlands.

## Lifted by wings of radicalism

With the ESTSS formally established, a stage was set for us newcomers to stand on the shoulders of European psycho-traumatology giants like Professors L. Weisaeth in Norway, L. Crocq in France and B. Gersons in the Netherlands. From these elevated positions, opportunities were created for members to develop and grow professionally and personally. Ours was an enthusiastic specialist field buzzing with a sense of being new. We were fresh, part of a movement carried forward by pioneers and taking shared ownership of investigations and accumulating clinical experience. At the start, there was something for everyone.

Radical and boundary breaking the ESTSS played a significant part in delivering its own extraordinarily gifted researchers, practitioners, and informed policies of prevention as well as post-incident response. Our strength derived not least from sustained healthy debate. Reflecting upon developments that have taken place over the last 20 years helps clarify some of the processes that have taken us to where we are now.

At the start we came together from our respective, often anonymous places of work where many of us had distanced ourselves from many of the professional orthodoxies that colleagues considered elemental to professional practice. We were united in our lack of fear and sometimes daring to think the unthinkable. We may not have known exactly where we were or our direction of travel but our momentum gave us leverage to carry on and on. We stepped beyond familiar horizons and made significant inroads where few had ventured before.

## The ESTSS phoenix: a bird of paradise, a swerve of illusions or a pyroclastic flow?

The society's successes are to be applauded as achievements of its members. While all are welcomed, there are also complementary considerations to take into account. Namely, that success also tends to carry a cost in its wake. An example is that while we were radical and boundary breaking, we are now largely mainstream. We are the new orthodoxies that are partly of our own making and some that are embedded in our work through considerations of prescriptive practice (e.g., treatment guidelines) borrowed from other specialisms. Anyone attending our conferences might ask what is particular or distinctive about our approach. Worldwide, is it not the case that the same themes run through conferences for other professions? It seems to me at times as if phoenixes have flocked and the winged formations comprise of many individuals with origins in other mythologies.

My applause for and appreciation of the ESTSS at 20 years gives me reason to feel proud of having made contributions to an area of professional development during two decades. But my praise and delight is not without qualification. I am increasingly concerned about the ESTSS collegiate being largely silent about those aspects of trauma that are most complex and complicated. A distinctive ESTSS voice that could be heard is muted. Ethics featured prominently in the conference programmes during early ECOTS. Now I wonder what happened to our interesting debates about moral considerations in trauma research and clinical practice. Have we ever considered if our insights about traumatic stress and trauma recovery are tools for torturers to inflict more serious and lasting damage on their victims? And what about aspects of trauma that we do not yet understand or for which there is not and never will be a single consensus, for instance, dropouts and non-response to interventions. I would welcome a greater intellectual curiosity within the ESTSS about the broader picture of trauma, e.g., why social support is immensely more powerful than therapy?

I would be reassured if ESTSS discourse were to a greater extent informed by an aspiration to nurture wisdom in the face of horror or if we foster restraint where fantasies of omnipotence easily take hold and the lifelong influences of memory. Herein lies a prospect for methodical studies that holds infinitely greater promise than subjecting human awareness to reductionist analysis. There is so much more to be said and learned about the human predicament, most especially as we emerge from crises, than constraints imposed by positivistic science (Robinson, 2010).

## Hitching a ride or stepping off?

The health status certificate awarded to ESTSS at two decades of maturity will have to take the vigour of debate and tolerance of diversity among its final criteria. In my view, the society is very good in some respect, and successes provide benchmarks for personal and professional purpose. Highly commendable is the new federal structure of our organisation. This resonates with the times in which we live. As our continent changes, so too do we go through personal, social and political transitions for which the ESTSS is an important catalyst and a source of support. I hitched a ride with this society two decades ago and I intend to stay.

Before concluding this personal flight of fancy, I would like to reflect on two linked thoughts that came to mind when asked to contribute to this presidents panel. I associate both with the aspirations and values we sought to promote when laying foundations for the ESTSS.

First a low point. It came while attending a treatment skills workshop for children and adolescents. A distressing transition from the already intolerable to the unbearable was mediated when techniques were considered for challenging survivors’ thoughts and mistaken beliefs about personal trauma. As I walked out I wondered, as I still do, how we dare confer on ourselves a presumption of superior knowledge about other people's trauma and their beliefs? My experience of survivors is that the more we ask about subjective experiences of trauma and survival, the greater the personal horrors of what has happened comes to light. A premise on which I hope the ESTSS is founded is that survivors know more about their trauma than any of us will, however, expert and professional we claim to be. I fear that questioning survivors’ thoughts and reactions in the way implied at the workshop reflects a wider and for me unwelcome propensity, as history repeats itself through us, of not wanting to know or be in denial.

Survivor narratives are almost invariably sanitised versions of the actual experience. In the telling, they protect themselves and us. Such restraint is functional in that it creates a safer psychological space for helpers to think about survivors’ ghastly memories and emotions. We should acknowledge that survivor's self-imposed censorship is their way of protecting those who offer support from the sometimes unspeakably awful. We as helpers may with good reason not want to know all details but to assert survivors have “wrong thoughts” is irrational. My personal agenda in psycho-traumatology has been to foster sustained awareness. I am disappointed by the degree to which our specialism has turned toward denial.

The second set of associations went as follows. The ESTSS track record must involve raising awareness of trauma, its sequelae, and effective interventions. Yet, I still struggle with a question of what the core matter or crucial ingredient of psycho-traumatology really is. Two answers come to mind. The first is that all considerations of trauma interfaces with human rights as they pertain to individuals right through to global matters. It is this practical and legal perspective, not theories or models, that should furnish the foundation for our field. I would welcome a shift in ESTSS, so human rights become its explicit focus.

For me, a second defining characteristic for a distinctive psycho-traumatology is the way trauma shows us how human memory works and that memory is central to human nature. I fear for the future of the ESTSS to the extent it condones attempts to interfere with or deny memory. An example is its tacit support of research, which explores possible ways of changing processes involved in memory recording, storage, or retrieval. Putatively, this is to lessen the traumatic impact of exposures. But attempts to interfere with nature and interfere with processes that have evolved to help humans adjust to prevailing circumstance, be they life threatening, traumatic or simply ordinary, strike me as imprudent. Lived experience is about more than memory, and impact occurs irrespective of whether we have full recall or not. For a decade or so, we have recognised that reactions evoked by trauma are often distressing and inconvenient and also have survival value (Shalev & Ursano, 2003) largely through influences exerted by memory.

## A phoenix readied for flight

I congratulate the ESTSS on its achievements over a period of 20 years. I am very proud to have been a part of its foundation and given shape to its formative years. We remain a young and youthful society with an impact factor vastly more impressive than its age suggests. As for the future, the gently tendered phoenix stands to gain its full wings and may yet soar to escape the gravitational pull of self-generated processes that will lead to its destruction. Psycho-traumatology is a difficult specialism for many reasons, and our best protection is to nurture a society which not only encourages but also tolerates critical peer review at all levels of its organisation. Numerically abundant and diverse in their variety, trauma gave rise to the ESTSS, remain its true subject and furnish its purpose. But trauma is like a volcano. In its dormant phases, it harbours a unique beauty. As it erupts, the processes envelop and destroy all that has been achieved. If this were to happen, it will be because of our failure to tender a finely balanced dynamic of formidable forces. On the other hand, if we gently tender the energies that bring life to the ESTSS and harness these to positive effect, the prospect of an onward flight is thrilling carried as it will be on hope, resolve, and a quest for truths that may be uncomfortable, but the journey we have started will never be completed. Travel with care and the view offered from the platform of trauma is like no other. In contrast to other specialisms, our field is not primarily a quest for certainty. Our strengths derive from how we accommodate and adjust uncertainties, ambiguities, and doubts. If the ESTSS tries to fly like Icarus, who came too close to the sun and could no longer fly, a catastrophic fall to earth is probable. Having the more modest aspirations of an emergent phoenix sustains cautious optimism for a future about which we know very little except that it is uncertain.
